# Effects of mindfulness meditation combined with progressive muscle relaxation on sleep disorders, anxiety, and depression in patients with sarcopenia undergoing hemodialysis

**DOI:** 10.3389/fpsyt.2025.1542028

**Published:** 2025-06-13

**Authors:** Yongyao Wu, Haojie Zhang, Lei Jiang, Zhiping Liu, Xinfei Li, Bei Guo, Junyuan Li, Pengjie Xu, Jiang Liu, Rizhen Yu

**Affiliations:** ^1^ Department of Nephrology, Ningbo Medical Center Lihuili Hospital, Ningbo, Zhejiang, China; ^2^ Urology & Nephrology Center, Department of Nephrology, Zhejiang Provincial People’s Hospital (Affiliated People’s Hospital), Hangzhou Medical College, Hangzhou, Zhejiang, China

**Keywords:** hemodialysis, mindfulness meditation, progressive muscle relaxation, uremic sarcopenia, sleep disorders

## Abstract

**Objective:**

Long-term physiological, psychological, economic, and lifestyle pressures make maintenance hemodialysis (MHD) patients prone to anxiety and depression. This study aims to evaluate the effects of mindfulness meditation combined with progressive muscle relaxation on sleep and emotional disorders in hemodialysis patients with sarcopenia.

**Methods:**

Patients with uremic sarcopenia and sleep issues were randomly divided into a control group (n=24) and an intervention group (n=25), with an average age of 45.4 ± 4.77 years and an average dialysis duration of 56.86 ± 17.48 months. The control group received standard treatment (hemodialysis three times per week, medication, health education, dietary guidance, and exercise recommendations), while the intervention group additionally underwent home-based mindfulness and relaxation training for at least 0.5 hours daily over 12 weeks. Sleep quality, emotional states, and quality of life were assessed using the Pittsburgh Sleep Quality Index (PSQI), Self-Rating Depression Scale (SDS), Self-Rating Anxiety Scale (SAS), and 36-Item Short Form Health Survey (SF-36). Data analysis included descriptive statistics, t-tests, analysis of covariance, Mann-Whitney U tests, and chi-square tests.

**Results:**

Before the intervention, there were no significant differences between the two groups in sleep, anxiety, depression, or quality of life (P>0.05). After the intervention, the intervention group showed significant improvements in all PSQI dimensions (e.g., sleep medication use, sleep quality, and sleep duration) (P<0.01), significant reductions in SAS and SDS scores (P<0.05), and significantly higher SF-36 scores compared to the control group (P<0.05).

**Conclusion:**

Mindfulness and muscle relaxation training can alleviate psychological stress, reduce skeletal muscle tension, promote muscle anabolism, and significantly improve sleep quality, anxiety, and depression in hemodialysis patients with sarcopenia. This approach is simple, unrestricted, and widely applicable, making it worthy of clinical promotion.

**Limitations:**

The study had a small sample size and a short follow-up period.

**Clinical trial registration:**

https://register.clinicaltrials.gov/, identifier NCT06261372.

## Introduction

The high incidence of uremic sarcopenia not only changes the lifestyle of patients, reducing their quality of life, but also increases the occurrence of cardiovascular complications, thereby significantly increasing the mortality rate of these patients and leading to a sharp increase in medical expenses (1). As a result of long-term physiological, psychological, economic, and lifestyle pressures, patients undergoing maintenance hemodialysis (MHD) are prone to anxiety, depression and sleep disorders. Studies have found that the incidence of anxiety and depression in patients undergoing MHD (53.33% and 46%, respectively) is much higher than the incidence in the general population (10% and 3%, respectively) (2). MHD patients in Northern China had high prevalence rates of depression (55.1%) and anxiety (25.9%) (3). The reported prevalence of insomnia in patients with chronic kidney disease ranges from 38 to 70% (4). Uremic sarcopenia affects physical health, contributing to a decline in overall well-being and quality of life, which may trigger or exacerbate anxiety and depression (5). Depression was causally related to decreased muscle mass, and declined muscle strength might lead to a higher risk of depression (6). A cross-sectional population-based study provides evidence of an association between sarcopenia and the prevalence of sleep disorders, with a negative correlation observed between the sarcopenia index and the odds ratio of sleep disorders (7). There are many studies on sarcopenia abroad. The harm it can cause to patients receiving MHD has been recognized, and the interventions for sarcopenia in patients receiving MHD have been studied. Research on sarcopenia in China is still in its infancy. Current studies have rarely focused on emotional problems in patients with sarcopenia receiving MHD and related interventions have been poorly investigated (8). Moreover, anxiety, depression, and sleep disorders are interrelated and mutually influence each other, becoming key factors that hinder patient recovery and affect prognosis, which severely impact patient quality of life. Currently, both domestically and internationally, the main intervention methods for patients with anxiety, depression, and sleep disorders are drug therapy. For uremic sarcopenia patients undergoing MHD, commonly used medications include anxiolytics such as benzodiazepines, antidepressants such as selective serotonin reuptake inhibitors (SSRIs), and hypnotics such as zolpidem and eszopiclone. Although drug treatment can shorten the time it takes for patients to fall asleep and improve the quality of their sleep, most hypnotic drugs have toxicity and tolerance issues, making them unsuitable for long-term use in patients undergoing MHD (9, 10). Therefore, finding a non-drug therapy has become an urgent issue. Mindfulness meditation training is a cognitive therapy that teaches patients to use or stimulate their inner strength through body awareness methods, improving their self-regulation and emotional cognition (11). Mindfulness meditation helps individuals better manage their thoughts and emotions by improving attention and concentration, and reduces psychological stress by increasing awareness and acceptance of automatic thinking, thereby improving sleep quality and reducing symptoms of depression and anxiety (12, 13). Progressive muscle relaxation training is a step-by-step relaxation training method (14) that has been applied abroad to improve the treatment of emotional and sleep disorders in patients with certain diseases (15). Progressive muscle relaxation training helps reduce symptoms of stress and anxiety and improve sleep quality by allowing individuals to learn to recognize and release physical tension (16). However, there is currently limited literature on patients with uremic sarcopenia undergoing MHD.

This study aims to explore the efficacy of mindfulness meditation combined with progressive muscle relaxation training in patients with uremic sarcopenia undergoing MHD, observe its effects on sleep disorders and anxiety and depression, and investigate its impact on patient quality of life. Despite the limitations of these sample sizes and intervention Settings, the study was designed to provide valuable insights into the potential efficacy of these interventions within a defined range. It is important to note that the present data represent secondary outcomes from our registered clinical trial (8) which primarily investigated the intervention’s effects on sarcopenia progression and nutritional status in this patient population.

## Methods

### Participants

The study is expected to last from August 14, 2023 to December 31, 2024. According to the diagnostic indicators of sarcopenia, 49 patients (36 men and 13 women) who had been diagnosed with sarcopenia were included in the study at Ningbo Li Huili Hospital’s Blood Purification Centre from August 15, 2022 to November 15, 2023. The participants had an average age of 45.4± 4.77 years, and the average duration of hemodialysis was 56.86 ± 17.48 months.

This study was a randomized controlled trial, divided into the intervention group and the control group. The Mos 36-item Short-Form Health Survey (SF-36) scale score of the subjects was the observed outcome index. According to literature review and pre-experimental results, the average SF-36 scale score of the control group was 72.38 ± 5.68 points, and the SF-36 scale score of the intervention was expected to decrease by 5.76 points. It has a 90% certainty. Calculate the sample size according to the following sample size calculation formula: n = 2(z_α_+z_β_)^2^*σ^2^/δ^2^, n=21 cases can be obtained. Considering 1:1 randomized grouping, that is, 21 subjects in each of the intervention group and the control group, and 10% of the cases of missing visits and refusing visits, at least 24 subjects in each of the intervention group and the control group will be required, and a total of at least 48 subjects will be included.

**Figure 1:**
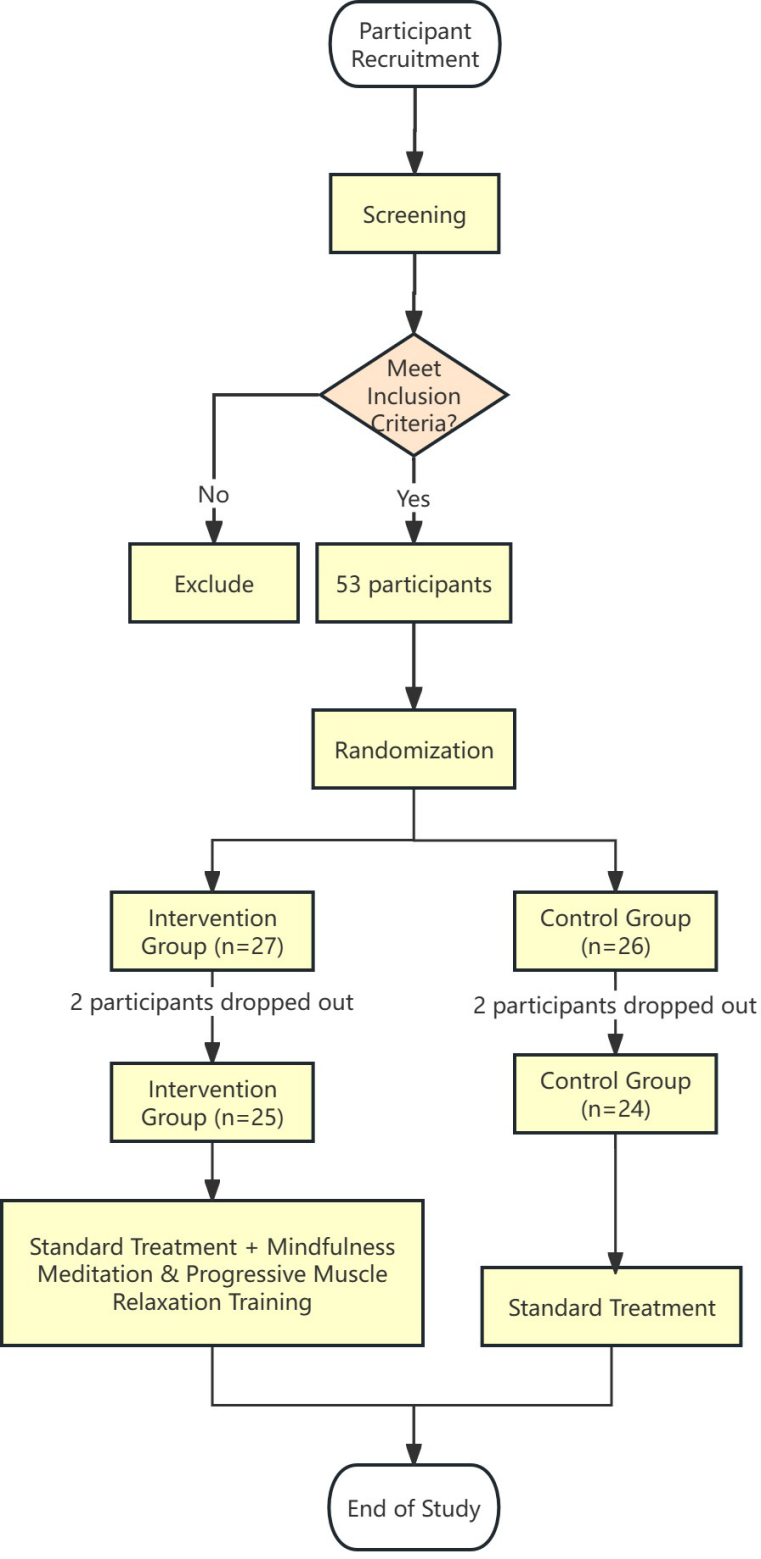
The flow chart of the participants.

A total of 53 patients from the blood purification center were screened, with two patients from each group withdrawing midway. Finally, 49 patients completed this trial study, achieving a completion rate of 92.45%. In the experimental group, both patients withdrew due to kidney transplantation. In the control group, one patient dropped out due to kidney transplantation and the other dropped out due to inability to complete the relevant questionnaire. The dropout rates for the intervention group and the control group were 7.4% and 7.6%, respectively. There were no significant differences in baseline indicators between the two groups. A total of 49 questionnaires were distributed in this study, and all 49 were collected, resulting in a 100% effective collection rate. The questionnaires collected detailed information including demographic data, medical history, baseline sleep quality, anxiety and depression levels, and quality of life assessments. There were no significant differences in baseline indicators between the two groups (**Table 1**). Participants were randomly divided into an intervention group (n = 25) and a control group (n = 24) using a random number table. SPSS software was used to generate a random number table, and corresponding numbers were given according to the order of the random number table before and after the admission of patients, and then according to odd and even numbers, odd numbers were used as the observation group, and even numbers were used as the control group. The participants were blinded to their group assignment and were instructed not to share any information about the intervention with the other group. The research leader made the assignment order; 2 deputy chief physicians and 1 psychologist participated in the registration; Interventions were assigned by two transfer nurses. The flow chart of the participants is shown in **Figure 1**.

**Table 1:** Characterization of study population (n =49).

Characterization of study population (n =49)	Intervention group (n=25)	Control group (n=24)	*X^2^/t/z*	*P*
Sex (%)			0.168	0.682
Men	19(76.0)	17(70.8)		
Women	6(24.0)	7(29.2)		
Age (year)	45.52 ± 4.43	45.46 ± 5.12	0.045	0.964
MHD duration (months)	59.16 ± 18.85	54.46 ± 15.98	0.940	0.352
Educational level (%)			1.380	0.240
Moderate and high	16(64.0)	19(79.2)		
Low	9(36.0)	5(20.8)		
Employment status (%)			0.698	0.404
Employed	9(36.0)	6(25.0)		
Unemployed	16(64.0)	18(75.0)		
Marital status (%)			0.525	0.469
Married	13(52.0)	10(41.7)		
Not married	12(48.0)	14(58.3)		
Income status (%)			1.041	0.308
High, moderate and above	12(48.0)	15(62.5)		
Low	13(52.0)	9(37.5)		
Medical insurance (%)			0.163	0.686
Good or moderate	17(68.0)	15(62.5)		
Poor	8(32.0)	9(37.5)		
Systolic pressure (mmHg)	151.72 ± 7.25	150.42 ± 7.16	0.633	0.530
Diastolic pressure (mmHg)	89.80 ± 10.41	86.88 ± 15.03	0.795	0.431
Dry weight (Kg)	58.9(57.5-66.8)	60.6(57.0-62.9)	-0.730	0.465
Etiology of ESRD (%)			7.106	0.418
purpura nephritis	3(12.0)	1(4.2)		
Gouty nephropathy	1(4.0)	2(8.3)		
Diabetes	2(8.0)	3(12.5)		
LN	2(8.0)	1(4.2)		
Obstructive nephropathy	0(0.0)	3(12.5)		
Membranous nephropathy	2(8.0)	1(4.2)		
Hypertension	1(4.0)	2(8.3)		
Other	14(56.0)	11(45.8)		

MHD, Maintenance Hemodialysis; ESRD, End-Stage Renal Disease; LN, Lupus nephritis.

This study was approved by the Ningbo Li Huili Hospital Ethics Committee, and informed consent was obtained from the participants (Ethical Approval No. KY2023SL204-01). The study’s purpose was explained, and participation was voluntary.

### Diagnostic criteria

The diagnostic criteria for anxiety, depression, and insomnia were based on the *Chinese Classification and Diagnostic Criteria of Mental Disorders* (17). The diagnostic criteria for muscle wasting were based on the *Muscle Wasting Diagnosis and Treatment Expert Consensus*, released by the Asian Working Group for Sarcopenia in 2019 (18).

### Inclusion and exclusion criteria

The inclusion criteria were as follows: (1) participants meeting the aforementioned diagnostic criteria for muscle wasting, insomnia, anxiety, and depression; (2) age ≥18 and <75 years, no major acute illness or exacerbation of a chronic condition, and able to perform daily activities without severe limitations; (3) good compliance and ability to cooperate well with healthcare providers; (4) hemodialysis duration of at least 3 months, with a KT/V ≥1.2; (5) absence of significant cardiovascular and cerebrovascular complications (e.g. severe heart failure, severe arrhythmias, angina). The exclusion criteria were as follows: (1) patients with contraindications for bioelectrical impedance analysis; (2) patients with psychiatric disorders other than anxiety and depression or severe cognitive or communication impairments.

### Control group

The control group received standard treatment, which included hemodialysis three times per week, medication, health education, dietary guidance, and exercise recommendations.

### Intervention group

In addition to the standard treatment received by the control group, the intervention group underwent mindfulness meditation combined with progressive muscle relaxation training during the intervals between hemodialysis sessions. The joint intervention team, comprising two meditation instructors, one psychological counselor, and one physician, possessed robust communication, coordination, and emergency response skills. All members receive regular training. The intervention team instructed patients to engage in self-practice for at least 0.5 hours per day for 12 weeks. To facilitate communication and support, we established a WeChat group to share mindfulness meditation and progressive muscle relaxation training materials, including audio tutorials and manuals, which were adapted from the sources cited in (19) and (20), respectively, and to remind patients of their daily practice. Additionally, patients could share feedback on their training experiences via the WeChat group. The joint intervention team monitored patients’ adherence to home practice and provided necessary support and guidance, as required.

### Progressive muscle relaxation training

The progressive muscle relaxation training intervention was implemented according to the specific guidelines outlined in the *Progressive Muscle Relaxation Training* manual by Smith (21). Patients were instructed to find a comfortable and quiet place to sit and begin with slow, controlled breathing through the nose and mouth. The relaxation of the entire body was divided into 16 groups, and patients were guided to perform alternating contractions and relaxations, with contractions lasting 10–15 seconds and relaxations lasting 15–20 seconds for each muscle group. Each muscle group relaxation exercise was repeated three times in the following order: one arm and then the other arm; one hand and then the other hand; shoulder muscles (one side and then the other); neck muscles, forehead, eyes, and scalp; jaw and mouth (skipping the tongue); chest, abdomen, lower back, and hips; one thigh and then the other thigh; one calf and foot and then the other calf and foot. Throughout the exercises, patients were instructed to focus on the sensation of blood flow in the corresponding areas of the body and guide the muscles to progressively relax. This structured approach aimed to induce relaxation and alleviate symptoms of anxiety and tension.

### Mindfulness meditation

After completing the muscle relaxation training, the specific intervention outlined in the standardized *Mindfulness Meditation* manual (22) was followed. Patients were instructed to choose the seated posture that was the most relaxing and stable, with options including crossed legs or sitting on a cushion or chair with the eyes closed. They were encouraged to maintain a straight spine, aligning the head with the spine, while maintaining a relaxed posture without becoming overly rigid. During the body scan, patients were guided to shift their attention to the body while breathing, identifying areas where they may feel slight tension or discomfort. They were instructed to observe these sensations, accept them, and as they continued to breathe, slowly move their attention away from those particular areas. In mindful breathing, patients concentrated their attention on their breath, taking deep breaths into the abdomen and exhaling slowly. They were encouraged to be aware of the inhalation and exhalation sensations, noticing the rhythm of breathing. During mindfulness meditation, patients focused their attention on becoming aware of thoughts, ideas, and emotions arising in the mind. They experienced these thoughts, ideas, and emotions as they came and went, guiding themselves to recognize negative thoughts without judgment or rejection. Patients prompted themselves to make appropriate responses when negative emotions surfaced and recognize the natural rhythm of breathing and the relaxation of the body. As they breathed, they noticed the body becoming calmer, more relaxed, and at ease. When ready, patients slowly opened both eyes on the exhalation. This process aimed to help patients become more mindful and aware of their thoughts, feelings, and bodily sensations while maintaining a relaxed and focused state.

Patients were instructed to record any questions or observations after each practice session. This record would be helpful for review and discussion in the following session, allowing for a deeper understanding of the mindfulness techniques.

### Observation metrics and methods

The indicators of both groups were assessed before and after the 12-week intervention. Includes:Standardized formula for sleep evaluation, The Pittsburgh Sleep Quality Index (PSQI), Mos 36-item Short-Form Health Survey (SF-36), Self-Rating Anxiety Scale (SAS) and Self Rating Depression Scale (SDS).

### Efficacy assessment criteria

The internationally standardized formula for sleep evaluation was used: sleep efficiency = actual sleep time/total time in bed × 100%. Clinical cure: symptoms disappear, and sleep efficiency is ≥75%; significant improvement: symptoms are relieved, and sleep efficiency is ≤65% and <75%; effective: symptoms improve, and sleep efficiency is ≤40% and <65%; ineffective: symptoms remain the same, and sleep efficiency is <40%. Total effective rate = (number of clinically cured cases + number of significantly improved cases + number of effective cases)/total cases × 100%.

Pittsburgh Sleep Quality Index (PSQI): This study used the Chinese version of Pittsburgh Sleep Quality Index, which is suitable for the Chinese population (23). It includes 19 self-rated and 5 other-rated items. However, the 19th self-rated item and the 5th other-rated item do not contribute to the scoring. The scale consists of seven scoring components: sleep quality, sleep latency, sleep duration, habitual sleep efficiency, sleep disturbances, use of sleep medications, and daytime dysfunction. Each component is scored on a 0–3 scale, and the total score ranges from 0 to 21. A score of ≤7 indicates improved sleep quality, whereas >7 indicates the presence of sleep disturbances, with higher scores indicating poorer sleep quality. The Chinese version of PSQI has a Cronbach’s α coefficient of 0.842 and a test-retest reliability coefficient of 0.809 (23).

Self-Rating Anxiety Scale (SAS) (24): Developed by Zung, this scale consists of 20 items, and each item is rated on a 4-point scale (1–4 points) based on feelings over the previous week. The cumulative scores of all items form the SAS total raw score, and the total raw score multiplied by 1.25 gives the SAS standard score. Cronbach’s α coefficient is 0.836, with a test-retest reliability coefficient of 0.832.

Self-Rating Depression Scale (SDS) (25): Also developed by Zung, this scale comprises 20 items, and each item is rated on a 4-point scale (1–4 points) based on feelings over the previous week. The cumulative scores of all items constitute the SDS total raw score, and the total raw score multiplied by 1.25 yields the SDS score. Cronbach’s α coefficient is 0.887, with a test-retest reliability coefficient of 0.821.

The Mos 36-Item Short-Form Health Survey (SF-36) is considered the universal gold standard for measuring health status, which includes 8 subscales and 36 items to assess the quality of life and health of a population in terms of both physical and mental health (25). Cronbach’s α coefficient is 0.858, with a test-retest reliability coefficient of 0.891. The SF-36 scale adopts the domestic unified standard for scoring. First, the rank of each item is compiled, and then the score of each dimension is added up. Finally, the formula is used for scoring. The basic steps include: the first step, scale item coding; The second step, the scale items score; The third step, all aspects of the scale of health scores and score conversion. Conversion score = Actual score - the lowest possible score for this aspect/the difference between the highest possible score and the lowest possible score for this aspect ×100 is converted so that the scores in each dimension are between 0 and 100, 0 is the worst, 100 is the best (26).

### Statistics

Statistical analysis was performed using IBM SPSS Statistics, version 17.0 (IBM Corp., Armonk, NY, USA). Continuous variables were first tested for normality using the Kolmogorov–Smirnov test. For variables that followed a normal distribution, baseline values were presented as mean ± standard deviation (SD) and compared using independent sample t-tests. For post-intervention values, analysis of covariance (ANCOVA) was used to adjust for baseline differences (pretest values) and calculate adjusted means and standard errors (SE). Continuous variables that did not follow a normal distribution were presented as median and interquartile range [M(P25–P75)] and compared using the Mann–Whitney U test. Categorical data were analyzed using the chi-square test. A P-value less than 0.05 was considered statistically significant.

## Results

### Comparison of clinical efficacy between the two groups

The total effective rate in the intervention group was 96.0%, whereas in the control group, it was 66.7%. The intervention group demonstrated a significant superiority over the control group (X^2^ = 5.207, p = 0.022) (**Table 2**). The compliance rate of this study is 100%.

**Table 2:** Comparison of clinical efficacy between two groups.

Group	n	Significant improvement	Improvement	better	Invalid	Total effective rate (%)
Intervention group	25	12	9	3	1	96.0
Control group	24	4	5	7	8	66.7
X^2^						5.207
*p*						0.022

### Comparison of the Pittsburgh Sleep Quality Index scores for various factors and total scores between the two groups

Before the intervention, no significant differences were observed between the intervention and control groups in total Pittsburgh Sleep Quality Index (PSQI) scores or scores for individual dimensions (all P > 0.05). After the 3-month intervention, the adjusted mean total PSQI score in the intervention group (7.59, standard error [SE] 0.34) was significantly lower than that in the control group (14.75, SE 0.36, P < 0.001). This statistically significant reduction in the total PSQI score indicates that the combined intervention of mindfulness meditation and progressive muscle relaxation was effective in improving overall sleep quality among participants in the intervention group. The intervention group also showed significant improvements in all PSQI dimensions compared with the control group, including sleep quality (1.29, SE 0.10 vs. 2.29, SE 0.11, P < 0.001), time to fall asleep (1.19, SE 0.08 vs. 2.14, SE 0.08, P < 0.001), sleep duration (1.22, SE 0.09 vs. 2.50, SE 0.10, P < 0.001), sleep efficiency (1.16, SE 0.18 vs. 2.13, SE 0.19, P < 0.001), sleep disturbances (1.24, SE 0.08 vs. 1.74, SE 0.08, P < 0.001), use of sleep medication (0.63, SE 0.09 vs. 1.97, SE 0.10, P < 0.001), and daytime dysfunction (0.89, SE 0.11 vs. 1.95, SE 0.12, P < 0.001) (**Table 3**). This indicates that the intervention was effective in enhancing the subjective perception of sleep quality, facilitating quicker sleep onset, increasing the total amount of sleep, improving the ratio of time spent asleep to the total time spent in bed, reducing the frequency and severity of disruptions during sleep, reducing the reliance on sleep aids, improving participants’ ability to function during waking hours.

**Table 3:** Comparison of PSQI scores between two groups with pretest value as a covariate.

Variable	Group	Pretest	Posttest (Unadjusted)	Posttest (Adjusted)	P value for interaction
Sleep quality	Intervention	2.44 ± 0.51	1.32 ± 0.56	1.29 (0.10)	<0.001
	Control	2.30 ± 0.56	2.26 ± 0.54	2.29 (0.11)	
Time to fall asleep	Intervention	2.28 ± 0.61	1.20 ± 0.50	1.19 (0.08)	<0.001
	Control	2.22 ± 0.42	2.13 ± 0.34	2.14 (0.08)	
Sleep time	Intervention	2.52 ± 0.51	1.24 ± 0.60	1.22 (0.09)	<0.001
	Control	2.43 ± 0.59	2.48 ± 0.51	2.50 (0.10)	
Sleep efficiency	Intervention	2.48 ± 0.87	1.36 ± 1.25	1.16 (0.18)	<0.001
	Control	2.00 ± 1.13	1.91 ± 1.24	2.13 (0.19)	
Sleep disorders	Intervention	1.60 ± 0.50	1.20 ± 0.50	1.24 (0.08)	<0.001
	Control	1.74 ± 0.45	1.78 ± 0.42	1.74 (0.08)	
Hypnotic drugs	Intervention	1.96 ± 0.54	0.64 ± 0.49	0.63 (0.09)	<0.001
	Control	1.91 ± 0.67	1.96 ± 0.64	1.97 (0.10)	
Daytime dysfunction	Intervention	1.88 ± 0.67	0.88 ± 0.67	0.89 (0.11)	<0.001
	Control	1.91 ± 0.60	1.96 ± 0.56	1.95 (0.12)	
PSQI Total Score	Intervention	15.16 ± 1.89	7.84 ± 2.59	7.59 (0.34)	<0.001
	Control	14.52 ± 2.29	14.48 ± 2.15	14.75 (0.36)	

PSQI, Pittsburgh Sleep Quality Index; The unadjusted and adjusted posttest values are expressed as mean ± standard deviation and mean (standard error), respectively.

### Comparison of self-rating anxiety scale and self-rating depression scale scores between the two groups

Before the intervention, there were no statistically significant differences in SAS and SDS scores between the intervention and control groups (all P > 0.05). After the 3-month intervention, the adjusted mean SAS score in the intervention group (36.29, SE 0.78) was significantly lower than that in the control group (51.73, SE 0.82, P < 0.001). Similarly, the adjusted mean SDS score in the intervention group (39.60, SE 0.96) was significantly lower than that in the control group (59.92, SE 1.00, P < 0.001). The data indicate that the intervention was successful in reducing levels of anxiety and depression among hemodialysis patients.

### Comparison of Mos 36-item Short-Form Health Survey scores between two groups of PSQI

Before the intervention, no statistically significant differences were observed in the SF-36 scores between the intervention and control groups (P > 0.05). After the intervention, the adjusted mean SF-36 score in the intervention group (78.06, SE 1.87) was significantly higher than that in the control group (71.50, SE 1.95, P = 0.019) (**Table 4**). The data is indicative of a meaningful enhancement in the overall health status and well-being of the participants who received the intervention.

**Table 4:** Comparison of psychological status between two groups with pretest value as a covariate.

Variable	Group	Pretest	Posttest (Unadjusted)	Posttest (Adjusted)	P value for interaction
SAS	Intervention	53.92 ± 5.76	36.28 ± 3.29	36.29 (0.78)	<0.001
	Control	52.78 ± 3.73	51.74 ± 4.39	51.73 (0.82)	
SDS	Intervention	60.00 ± 5.14	39.63 ± 4.55	39.6 (0.96)	<0.001
	Control	62.22 ± 5.91	59.96 ± 4.88	59.92 (1.00)	
SF-36	Intervention	68.40 ± 9.24	77.96 ± 7.34	78.06 (1.87)	0.019
	Control	69.35 ± 8.06	71.61 ± 11.24	71.50 (1.95)	

SAS, Self-Rating Anxiety Scale; SDS, Self-Rating Depression Scale; SF-36, 36-Item Short Form Health Survey. The unadjusted and adjusted posttest values are expressed as mean ± standard deviation and mean (standard error), respectively.

## Discussion

The present study investigated the efficacy of combining mindfulness meditation with progressive muscle relaxation training to improve sleep disorders, anxiety, and depression in patients with sarcopenia undergoing MHD. This secondary analysis of our registered RCT (8) demonstrates that 12 weeks of mindfulness-based relaxation training effectively improved sleep quality and emotional states in hemodialysis patients with sarcopenia. The results indicate a significant improvement in the intervention group compared with the control group across various measures. In Zhu et al. ‘s study, wuling capsules exhibited good efficacy and safety in the treatment of sleep disorders, anxiety, and depression in patients receiving maintenance hemodialysis (27). cognitive behavioral group therapy for insomnia (CBGT-I) has a similar effect. CBGT-I is effective in reducing depression and anxiety in addition to improving sleep quality and general psychological health in hemodialysis patients (28). However, comparisons were not possible due to different assessment tools used in the studies and different patient baselines, the efficacy of combining mindfulness meditation with progressive muscle relaxation training will be further explored in the future.

Mindfulness meditation training integrates principles of mindfulness therapy and meditation, emphasizing conscious awareness, present-moment attention, and non-judgment. Through techniques such as body scanning and mindfulness breathing, this training enhances focus and emotional regulation (29). This approach not only improves emotional well-being but also has a significant impact on sleep quality, effectively alleviating symptoms of depression and anxiety. In addition to mindfulness meditation, this intervention incorporates progressive muscle relaxation training (30). Grounded in the theory of reciprocal inhibition, this relaxation therapy balances the autonomic nervous system, leading to reduced anxiety, improved sleep quality, and the rapid induction of relaxation (31). By lowering skeletal muscle tension and promoting muscle synthesis metabolism, progressive muscle relaxation aids in the recovery of skeletal muscle function. Moreover, it complements the psychological relaxation induced by mindfulness meditation (32). Together, these two approaches synergistically enhance patients’ physical and psychological well-being, offering a comprehensive intervention for addressing conditions such as sarcopenia.

The positive effects of mindfulness meditation and progressive muscle relaxation training in alleviating anxiety and depression, including anxiety and depression caused by disease, have been widely reported (29–32). However, there are fewer reports on their combined use in interventions for patients with sarcopenia undergoing MHD. In this study, mindfulness meditation combined with progressive muscle relaxation training was applied to patients with anxiety, depression, and sleep disorders undergoing MHD. The results revealed that the intervention group’s PSQI total score and PSQI scores for each item were better than those of the control group, with statistical significance. In the items related to sleep quality, sleep duration, and the use of sleep medication, the statistical differences were particularly significant. This indicates that mindfulness meditation combined with progressive muscle relaxation training has a positive effect on insomnia, particularly in reducing the use of sleep medication. It can effectively improve the sleep status of patients, especially in terms of sleep quality and sleep duration. After 3 months, the SAS and SDS scores in the intervention group decreased significantly, and the SF-36 scores increased significantly. By contrast, the control group exhibited no significant differences before or after treatment. This suggests that this training method can effectively improve the anxiety and depression status of patients and has a positive impact on the quality of life of patients undergoing MHD.

This study represents a pioneering effort to address sleep disorders in patients with end-stage renal disease and sarcopenia, a topic that has received limited attention in the literature. Our findings suggest that the combined approach of mindfulness meditation and progressive muscle relaxation training can effectively enhance sleep quality in these patients. This aligns with previous research demonstrating the positive impact of mindfulness meditation on sleep disturbances associated with various physical illnesses, including hypertension, cancer, and stroke (33–35). The mechanisms underlying improvements in sleep quality through mindfulness meditation training are still a subject of debate. Existing studies propose several potential mechanisms, including anti-hyperarousal effects, psychosocial factors, and neurochemical changes within the brain. Lin et al. (36) conducted research indicating that mindfulness meditation can regulate patient emotions, thereby influencing the timing and expression of emotions, as well as cognitive processes and emotional responses, which may contribute to reducing sleep hyperarousal. Mindfulness-based stress reduction has been demonstrated to enhance emotional regulation by increasing the sensitivity of the left prefrontal cortex, consequently reducing negative emotions and improving sleep quality. Additionally, mindfulness therapy may facilitate hypothalamic neural conduction, promoting sleep induction and reducing overall sympathetic nervous system activity and psychological arousal (37). Studies investigating mindfulness therapy have reported notable changes in neurotransmitter levels following intervention. After an 8-week mindfulness program, participants exhibited decreased levels of adrenocorticotropic hormone, norepinephrine, and adrenaline compared with the control group, alongside increased levels of dopamine and melatonin. These findings suggest that mindfulness therapy has a significant impact on the sympathetic adrenal medullary system (38).

### Strengths and limitations

The research has the following advantages: First, the study introduces a novel approach by combining mindfulness meditation with progressive muscle relaxation as an intervention for patients with sarcopenia undergoing hemodialysis. This dual approach addresses both the psychological and physical aspects of the condition. Second, the study focuses on a specific and often overlooked population—patients with uremic sarcopenia and sleep issues undergoing hemodialysis—providing tailored interventions that can improve their quality of life.

However, this study has some limitations, including a relatively small sample size with short follow-up period and the need for further refinement of the standardized procedures and training duration for mindfulness meditation. In future studies, larger samples will be used to verify the results of this study, the follow-up time will be extended, and the standardized procedures and training time of intervention will be further improved.

## Conclusion

In conclusion, mindfulness meditation combined with progressive muscle relaxation training can effectively improve sleep disorders, anxiety, and depression in patients with sarcopenia undergoing MHD. It can also enhance their overall quality of life. This approach offers advantages such as simplicity, lack of restrictions, and broad applicability, making it worthy of clinical promotion. The results of this study provide healthcare providers with an evidence-based, non-invasive way to improve sleep quality in patients with sarcopenia undergoing MHD, which could reduce reliance on sleep medications and their potential side effects.

## Data Availability

The original contributions presented in the study are included in the article/supplementary material. Further inquiries can be directed to the corresponding authors.
